# Epidemiological and microbial trends of infective endocarditis in western Norway: a 7-year prospective observational study

**DOI:** 10.1186/s12879-024-09596-3

**Published:** 2024-07-17

**Authors:** Stina Jordal, Øyvind Kommedal, Rune Haaverstad, Sahrai Saeed, Einar Skulstad Davidsen, Pirjo-Riitta Salminen, Karl Ove Hufthammer, Bård Reiakvam Kittang

**Affiliations:** 1 Section of Infectious Diseases, Department of Medicine, Haukeland University Hospital, Bergen, Norway; 2 Department of Clinical Science, University of Bergen, Bergen, Norway; 3 Department of Microbiology, Haukeland University Hospital, Bergen, Norway; 4 Section of Cardiothoracic Surgery, Department of Cardiology, Haukeland University Hospital, Bergen, Norway; 5 Department of Cardiology, Haukeland University Hospital, Bergen, Norway; 6 Centre for Clinical Research, Haukeland University Hospital, Bergen, Norway; 7 Department of Medicine, Haraldsplass Deaconess Hospital, Bergen, Norway

**Keywords:** Infective endocarditis, *Staphylococcus aureus*, Enterococci, Prosthetic material

## Abstract

**Background:**

In this prospective, observational study, we aimed to investigate epidemiologic and microbial trends of infective endocarditis in western Norway.

**Methods:**

Clinical and microbiological characteristics of 497 cases of infective endocarditis from 2016 through 2022 were investigated. Categorical data were analysed using Chi-squared tests. Survival data were analysed using multiple Cox regression and reported using hazard ratios.

**Results:**

The mean age was 67 years, and 74% were men. The annual incidence rates varied from 10.4 to 14.1 per 100,000 inhabitants per year. Infective endocarditis on native valves was observed in 257 (52%) of the cases, whereas infective endocarditis on prosthetic valves and/or cardiac implantable electronic devices was observed in 240 (48%) of the cases: infection on surgically implanted bioprostheses was observed in 124 (25%) of the patients, infection on transcatheter aortic valve implantation was observed in 47 (10%) patients, and infection on mechanical valves was observed in 34 (7%) cases. Infection related to cardiac implantable electronic devices was observed in a total of 50 (10%) cases.

*Staphylococcus aureus* and viridans streptococci were the most common microbial causes, and isolated in 145 (29%) and 130 (26%) of the cases, respectively.

Enterococcal endocarditis showed a rising trend during the study period and constituted 90 (18%) of our total cases of infective endocarditis, and 67%, 47%, and 26% of the cases associated with prosthetic material, transcatheter aortic valve implantation and cardiac implantable electronic devices, respectively.

There was no significant difference in 90-day mortality rates between the native valve endocarditis group (12%) and the group with infective endocarditis on prosthetic valves or cardiac implants (14%), *p* = 0.522. In a model with gender, age, people who inject drugs, microbiology and type of valve affected, only advanced age was significantly associated with fatal outcome within 90 days.

**Conclusions:**

The incidence of infective endocarditis, and particularly enterococcal endocarditis, increased during the study period. Enterococci appeared to have a particular affinity for prosthetic cardiac material. Advanced age was the only independent risk factor for death within 90 days.

**Supplementary Information:**

The online version contains supplementary material available at 10.1186/s12879-024-09596-3.

## Background

Infective endocarditis (IE) has a considerable burden on critical healthcare resources with estimated in-hospital mortality rates up to 18% [1]. The epidemiology is changing, probably due to increasing age in the IE- population, a lower threshold for heart valve surgery, and increased diagnostic awareness [1, 2]. During the last decade, the prevalence of enterococcal endocarditis (EE) has increased, the importance of which is also reflected in the revised Duke-ISCVID criteria, where the identification of *Enterococcus faecalis* is proposed as a major criterion for IE [2–5]. Our previous study, retrospectively covering IE in western Norway during the period 1996–2015, showed increasing overall annual incidence rates of IE from 4.6 – 7.4 per 100.000 inhabitants and a significant increase in enterococcal endocarditis (EE) from 4 to 13% during the two decades [6].

To follow the most recent IE trends, explore correlations in more detail, and propose possible explanations for the observed changes, we prospectively collected and investigated comprehensive clinical and microbiological data from a total of 497 patients treated for IE in our region in western Norway from 2016 through 2022. The main aims of the present study were to investigate clinical features and microbial causes of IE, risk factors for fatal outcome, and predictors of native valve endocarditis (NVE) and prosthetic valve endocarditis (PVE), including IE on transcatheter aortic valve implantation (TAVI) and cardiac implantable electronic devices (CIED).

## Materials and methods

### Study setting and population

Clinical and microbiological data from 497 IE cases in the period 2016 through 2022 were prospectively collected. All patients were recruited from the tertiary care hospital Haukeland University Hospital (HUH) or the secondary care hospital Haraldsplass Deaconess Hospital (HDH), both located in the municipality of Bergen, western Norway. The population of the catchment area increased from 513,038 to 547,458 during the study period [7]. In addition, HUH has a regional responsibility for IE-patients with a complicated clinical course requiring multidisciplinary evaluation by infectious disease specialists, cardiologists and cardiothoracic surgeons for the entire Health Region West, comprising 1.1 million inhabitants [7]. To minimize referral bias, only patients belonging to the catchment areas of HUH or HDH were included in the incidence calculations whereas all patients were included in the statistical analysis to reflect the patient composition of the total sample.

All patients included were ≥ 18 years of age. Cases were identified upon admission and included when the diagnosis of definite or possible IE according to the modified Duke criteria and the 2015 ESC guidelines, was established [8, 9]. Both patients with definite and possible IE were included, as patients in both groups received similar treatment, in line with the national guidelines for antimicrobial therapy of endocarditis in Norway [10]. Repeated IE included both relapse and reinfection. Sample collection procedures and antibiotic treatment practices remained unchanged during the study period. The study was approved by the Regional Committee for Medical Research Ethics Western Norway (REK Vest, approval no. 2015/ 1170). Written, informed consent was obtained from all patients.

All-cause mortality at 90 days was chosen as the main endpoints since death within this relatively wide timeframe was considered the most sensible parameter for the estimation of IE-related death, in line with previous publications [11, 12]. Secondary aims were predictors of NVE and PVE including CIED-IE and factors affecting the outcome in these two groups (NVE versus PVE/CIED-IE).

### Microbial isolates

All microbial isolates were cultured from blood, and matrix-assisted laser desorption/ionization – time of flight (Maldi-TOF MS) was used for microbial speciation. In patients undergoing surgery, the excised valves were routinely cultured. All culture-negative valves were investigated using broad-range amplification of the bacterial 16S rRNA directly from the sample DNA, followed by Sanger sequencing (direct 16S rRNA sequencing).

### Statistics

Data were analysed using IBM SPSS Statistics, Version 29.0.2.0 (Armonk, NY: IBM Corp). Continuous variables are presented as mean ± standard deviation and categorical variables as proportions. Groups were compared by Student’s t-test for continuous variables and Chi-squared tests for categorical variables. Survival was analysed by using univariable and multivariable Cox regression models and the results are reported using the hazard ratio (HRs) with 95% confidence intervals (CIs). A two-sided *p*-value ≤ 0.05 was considered statistically significant.

## Results

### Clinical characteristics

Baseline characteristics and outcomes are displayed in Table 1. The mean age was 67 years, and 366 (74%) of the patients were male. Eighty-one patients (16%) were people who inject drugs (PWID). Prior IE was documented in 85 patients (17%), diabetes mellitus in 75 (15%), dental visits within the last 6 months prior to admission as reported by the patients in 53 (11%) and 14 (3%) of the patients were treated with haemodialysis. A total of 50 patients (10%) with a mean age of 61 years, were referred from the tertiary care centre Stavanger University Hospital. In this cohort, 20 (40%) patients had a PVE/CIED-IE.

**Table 1:** Clinical characteristics, bacterial aetiology, and outcomes for the total IE group, NVE and PVE/CIED-IE in western Norway in the period 2016–2022

Variable	Category	Total *N* = 497 (%)	NVE *N* = 257(%)	PVE + CIED *N* = 240 (%)	*p*-value
Age, years	Mean (SD)	67 (18)	62 (19)	72 (14)	< 0.001
Gender	Male, n (%)	366 (74)	177 (69)	189 (79)	0.012
	Female, n (%)	131 (26)	80 (31)	51 (21)	
Total mortality	30-day, n (%)	42 (9)	23 (9)	19 (8)	0.679
	90-day, n (%)	65 (13)	31 (12)	34 (14)	0.522
	Overall, n (%)	192 (39)	90 (35)	102 (43)	0.087
Regional referrals^a^	Yes, n (%)	50 (10)	30 (60)	20 (40)	0.304
Age, years regional referrals	Mean (SD)	61 (17)	58 (16)	65 (18)	0.148
In-hospital days	Mean (SD)	29 (19)	28 (19)	31 (19)	0.063
Predisposing conditions	PWID, n (%)	81 (16)	61 (24)	20 (8)	< 0.001
	Haemodialysis, n (%)	14 (3)	8 (3)	6 (3)	0.680
	Diabetes mellitus, n (%)	75 (15)	34 (13)	41 (17)	0.230
	Dental procedures, n (%)	53 (11)	35 (14)	18 (8)	0.028
	Prior endocarditis, n (%)	85 (17)	29 (11)	56 (23)	< 0.001
Septic embolization	Yes, n (%)	341 (69)	189 (74)	152 (63)	0.014
TTE at baseline	Ejection fraction^b^, cm (SD)	56^c^ ( 11)	58^d^ (10)	53^e^ (12)	< 0.001
PET-CT used in diagnostics^f^	Infection confirmed, n (%)	67 (13)	5 (2)	62 (26)	< 0.001
Definite IE^g^	Yes, n (%)	390 (78)	209 (81)	181 (75)	0.109
Repeated IE	1 admission, n (%)	442 (89)	235 (91)	207 (86)	0.065
	≥ 2 admissions, n (%)	55 (11)	22 (8.6)	33 (14)	
Surgery performed	Bioprosthesis, n (%)	131 (26)	83 (32)	54 (23)	< 0.001
	Mechanical prosthesis, n (%)	24 (5)	24 (9)	0	
Known malignancy	Yes, n (%)	83 (17)	33 (13)^h^	50 (21)^i^	0.017
New malignancy^j^	Yes, n (%)	17 (3)	9 (4)	8 (4)	0.918
Microbiology	*Staphylococcus aureus*^k^*,* n (%)	145 (29)	96 (37)	49 (20)	< 0.001
	Viridans streptococci^l^, n (%)	130 (26)	76 (30)	54 (23)	0.073
	Enterococci^m^, n (%)	90 (18)	30 (12)	60 (25)	< 0.001
	Non-viridans streptococci^n^, n (%)	35 (7)	16 (6)	19 (8)	0.462
	No growth, n (%)	36 (7)	17 (7)	19 (8)	0.576
	Other^o^, n (%)	61 (12)	22 (9)	39 (16)	0.009

*Abbreviations*: *IE* Infective endocarditis, *NVE* Native valve endocarditis, *PVE* Prosthetic valve endocarditis, *CIED* Cardiac implantable electronic devices, *PWID* People who inject drugs, *TAVI* Transcatheter aortic valve implantation, *SD* Standard deviation, *TTE* Transthoracic echocardiography, *SUS* Stavanger university hospital

^a^Referrals from the other tertiary care centre in the Health Region West; Stavanger university hospital

^b^Ad modum Teicholz

^c^Documented in 307 cases

^d^Documented in 168 cases

^e^Documented in 139 cases

^f^PET-CT was mainly performed in addition to TEE, and only 8 patients had PET-CT and not TEE performed. Of these, PET-CT confirmed the diagnosis in three patients

^g^According to modified Duke criteria + ESC 2015 guidelines

^h^Prostate/urogenital *n* = 14, gastrointestinal *n* = 7, breast *n* = 5, haematological *n* = 2, others *n* = 5

^i^Prostate/urogenital *n* = 23, gastrointestinal *n* = 13, breast *n* = 3, haematological *n* = 4, others *n* = 7

^j^Prostate *n* = 2, gastrointestinal *n* = 8, others *n* = 7

^k^Methicillin-resistant *S. aureus* *n* = 1

^l^*Strep. mitis/oralis* *n* = 56, *Strep. sanguinis* *n* = 26*, Strep. parasanguinis* *n* = 6, *Strep. gordonii* *n* = 11, *Strep. salivarius* *n* = 11, *Strep. mutans* *n* = 7, *Strep. pneumoniae* *n* = 7, *Strep. constellatus* *n* = 2*, Strep. cristatus* *n* = 2, *Strep. intermedius* *n* = 1, *Strep. vestibularis* *n* = 1

^m^*Enterococcus faecalis* *n* = 83, *E. faecium* *n* = 6, *E. hirae* *n* = 1

^n^*Strep. dysgalacticae* *n* = 18, *Strep. agalacticae* *n* = 7, *Strep. pyogenes* *n* = 2, *Strep. bovis/equinus* complex *n* = 8

^o^Including *Staph. epidermidis* *n* = 14, *Staph. hominis* *n* = 3, *Staph. lugdunensis* *n* = 4, HACEK *n* = 14, *Candida spp. n* = *5*, Enterobacterales n = 3, *Rothia* spp. *n* = 2, *Granulicatella* spp. *n* = 2, *Abiotrophia defective n* = *2*, *Bacillus cereus n* = *1*, *Cutibacterium acnes* *n* = 4, *Brucella melitensis* *n* = 1*, Erysipelothrix rhusiopathiae* *n* = 1, *Gemella haemolysans* *n* = 1, *Aerococcus urinae* *n* = 1, *Coxiella burnetii* *n* = 3

**Fig. 1:**
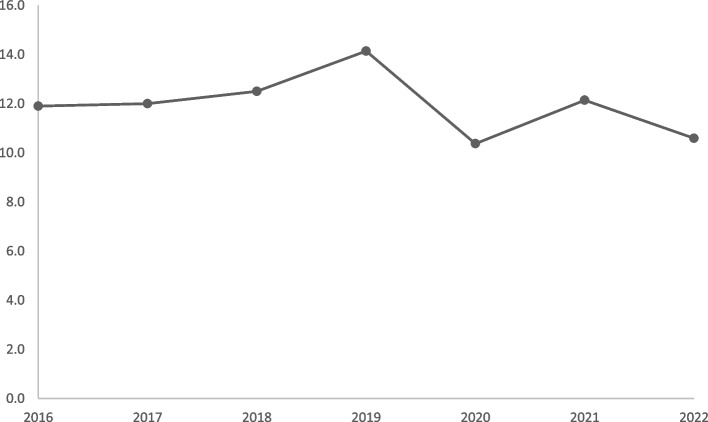
Incidence of infective endocarditis per 100 000 persons per year in western Norway in the period from 2016–2022

As shown in Fig. 1, the annual incidence rates varied from 10.4–14.1 per 100 000 inhabitants per year in the period 2016–2022, with the lowest incidence in 2020.

**Fig. 2:**
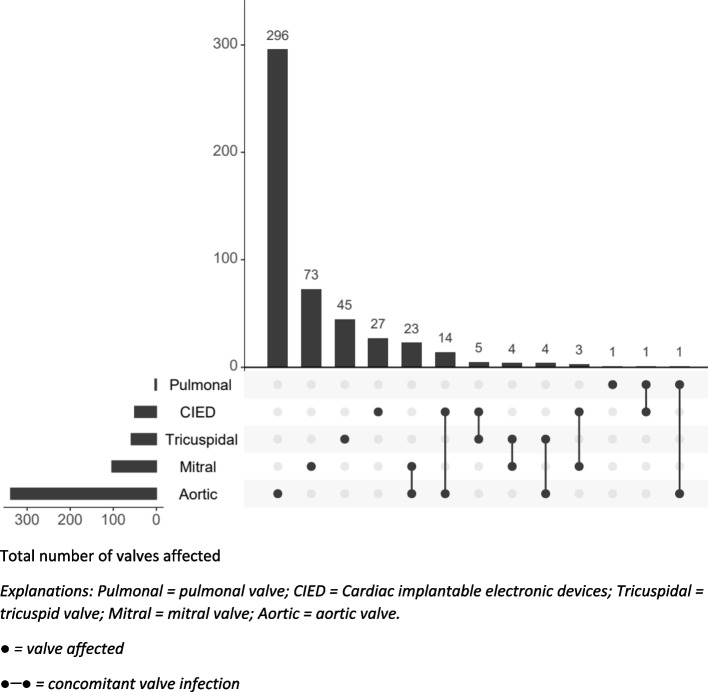
Distribution of infective endocarditis on different valves in western Norway in the period 2016–2022

The aortic and mitral valves were affected in 338 (68%) and 103 (21%) of the patients, respectively. Among the aortic endocarditis cases were 23 patients with concomitant aortic and mitral valve infection, 14 patients with infection both on the aortic valve and a CIED, four patients with infection both on the aortic and tricuspid valve, and one patient with concomitant aortic and pulmonary valve infection. The tricuspid valve was affected in 58 (12%) patients whereas pulmonal valve endocarditis was evident in only three patients. Based on findings from echocardiography or positron emission tomography-computed tomography (PET-CT), several combinations of concomitant valve infections and combinations of valve and CIED infection were observed (Fig. 2).

NVE accounted for 257 (52%) of the cases, whilst 240 (48%) of the patients had PVE/CIED-IE. The mean age was significantly lower in the NVE than in the PVE/CIED-IE group (67 ± 18 vs. 72 ± 14, *p *< 0.001). Females accounted for 80 (31%) of the NVE patients and 51 (21%) of the PVE/CIED-IE patients (*p* = 0.012).

In the NVE group, 33 patients (13%) had a history of active malignancy detected within the last 5 years preceding admission, whilst 50 patients (21%) in the PVE/CIED-IE group had a similar history of malignancy (*p* = 0.017). Prostate/urogenital cancer and gastrointestinal cancer were most common in both groups (Table 1). The prevalence of newly detected malignancies during the hospital stay was 4% of patients in both groups (*p *= 0.918); the majority of which were gastrointestinal cancers.

IE on surgically implanted bioprostheses was identified in 124 (25%) patients, whereas TAVI-IE was identified in 47 (10%), and IE on a mechanical valve was identified in 34 (7%) patients. CIED-IE was identified in a total of 50 (10%) cases; in 24 (48%) of these, the infection was restricted to the CIED. Among the remaining cases of CIED-IE there was concomitant involvement of either a prosthetic (*n* = 15) or native (*n* = 11) valve. The mean time from TAVI-procedure to the development of IE was 1.4 years (± 1.2 years), cefazolin was used for antibacterial prophylactic treatment, and given 15–30 min prior to the procedure.

A total of 160 patients (32%) had surgical treatment for IE. Of these, 131 (81%) patients received biological prostheses, whereas mechanical prostheses were preferred in 24 patients (15%) (Table 1). Mitral valve repair was performed in 16 (3%) of the cases, tricuspid valve repair in two, whereas 29 patients (6%) had an aortic vascular graft implanted concomitantly with a valve.

### Mortality rates

Thirty-day mortality was 42 (9%) whereas 65 patients (13%) died within 90 days after admission. A total of 192 patients (39%) died during the study period, with a mean follow-up of 3.2 years (± 2.3).

There was no significant difference in 90-day mortality rates between the NVE group and the PVE/CIED-IE group (12% versus 14%, *p* = 0.522) (Table 1).

A Cox regression analysis of mortality was performed, with administrative censoring at day 90 and surgery with mechanical or biological valve as a time-dependent covariate. In a model with gender, age, PWID, microbiological findings and type of valve affected, only high age (HR 1.04 per year, 95% CI 1.02–1.07, p < 0.001), was statistically significantly associated with fatal outcome (Table 2).

**Table 2:** Results from Cox regression analysis^a^ of risk factors for fatal outcome in 497 patients treated for IE in western Norway in the period 2016 – 2022

Model	Unadjusted			Fully adjusted		
**Variables**	**HR**	**95% CI**	***p***-value	**HR**	**95% CI**	***p***-value
Sex (female vs. male)	0.57	0.35–0.95	0.030	0.64	0.39–1.08	0.092
Age (per year)	1.03	1.01–1.04	0.006	1.04	1.02–1.07	< 0.001
PWID (yes vs. no)	0.83	0.41–1.67	0.593	2.58	0.87–7.62	0.087
*Staphylococcus aureus* (yes vs. no)	1.26	0.75–2.10	0.378	1.62	0.47–5.55	0.443
Viridans-streptococci (yes vs. no)	0.91	0.51–1.62	0.741	1.10	0.32–3.84	0.879
Enterococci (yes vs. no)	0.77	0.39–1.50	0.438	0.96	0.26–3.53	0.947
Non-viridans streptococci (yes vs. no)	1.90	0.91–3.98	0.090	2.57	0.68–9.75	0.166
Others (yes vs. no)	0.72	0.31–1.68	0.450	0.97	0.24–3.90	0.966
Valve affected (NVE vs. PVE)	0.95	0.58–1.55	0.822	0.92	0.54–1.57	0.767
Surgery performed						
Biological valve (yes vs. no)	0.49	0.22–1.09	0.080	0.71	0.31–1.63	0.422
Mechanical valve (yes vs.no)	0.34	0.05–2.45	0.283	1.07	0.13–8.57	0.422

*Abbreviations*: *IE* Infectious endocarditis, *HR* Hazard ratio, *CI* Confidence interval, *PWID* People who inject drugs, *NVE* Native valve endocarditis, *PVE* Prosthetic valve endocarditis

^a^With surgery with mechanical/biological valve as time-dependent covariate. Administrative censoring 90 at days (censored 432 (87%), events 65 (13%) patients)

### Microbiological findings

*Staphylococcus aureus* (*S. aureus*) was the most common bacterium, identified in 145 (29%) of the cases, followed by viridans streptococci in 130 (26%), enterococci in 90 (18%) and non-viridans streptococci in 35 (7%) of the cases. Thirty-six (7%) of the cases were culture negative (Table 1). Among the 90 cases of EE, *Enterococcus faecalis* was identified in 83 (92%), *Enterococcus faecium* in 6 (7%) and *Enterococcus hirae* in one case.

Enterococci were identified more often in PVE/CIED-IE than in NVE (25% vs. 12%, *p* < 0.001), whereas *S. aureus* was more frequently associated with NVE than PVE/CIED (38% vs. 20%, *p* < 0.001). In total, 67% of the IE cases related to cardiac prostheses and/or intracardial electronic devices were caused by enterococci.

The prevalence of different bacteria according to type of valve affected—categorized as native valve, surgically implanted bioprostheses, TAVI, mechanical prostheses and CIED are shown in Table 3.

**Table 3:** Microbial aetiology according to type of infected valves for patients with NVE, PVE and CIED IE in western Norway from 2016–2022

	**Native valve** ***n***= 257 (%)	**Surgical** **bioprosthesis**^**a**^ ***n*** = 124 (%)	**TAVI**^**b**^ ***n*** = 47 (%)	**CIED**^**c**^ ***n*** = 35 (%)	**Mechanical valve**^**d**^ ***n*** = 34 (%)	***p***-value^**e**^
*Staphylococcus aureus,* n (%)	96 (37.4)	23 (18.5)	6 (12.8)	14 (40.0)	6 (17.6)	< 0.001
Viridans streptococci, n (%)	76 (29.6)	37 (29.8)	10 (21.3)	1 (2.9)	6 (17.6)	0.007
Enterococci, n (%)	30 (11.7)	25 (20.2)	22 (46.8)	9 (25.7)	4 (11.8)	< 0.001
Non-viridans streptococci,n (%)	16 (6.2)	9 (7.3)	1 (2.1)	4 (11.4)	5 (14.7)	0.193
Other microbes, n (%)	22 (8.6)	21 (16.9)	7 (14.9)	3 (8.6)	8 (23.5)	0.032
No growth, n (%)	17 (6.6)	9 (7.3)	1 (2.1)	4 (11.4)	5 (14.7)	0.222

*Abbreviations*: *NVE* Native valve endocarditis, *PVE* Prosthetic valve endocarditis, *CIED* Cardiac implantable electronic devices, *IE* Infective endocarditis, *TAVI* Transcatheter aortic valve implantation

^a^Including 9 patients with CIED and biological prosthesis and one with mitraclip

^b^Including 4 patients with CIED in combination with TAVI

^c^Including 11 patients with native valve and CIED

^d^Including 2 patients with CIED in addition to a mechanical valve

^e^Chi-square test or Fisher’s exact test when appropriate

Enterococci were more frequently identified in TAVI-IE (47%) than in the other groups (12%–26%). *Staphylococcus aureus* was more often associated with NVE (37%) and CIED (40%) than the other groups (13%–19%). Viridans streptococci were more often identified in NVE (30%) and in surgically implanted bioprotheses (30%) than in the other groups (3%-21%).

Microbial growth from the excised valves was obtained in 22 (14%) of the 160 operated patients, whereof enterococci constituted 9 (41%). Mean time from admission to surgery was shorter in the enterococcal group than in the *S. aureus* group (10 days ± 13 vs. 19 days ± 28, *p* = 0.022). All bacterial isolates cultured from the valves had already been recovered from blood cultures. Bacterial DNA was detected in 136 (85%) valve biopsies and provided microbial identification for eight culture negative cases.

A total of 55 patients were admitted with repeated IE. Relapse within 6 months occurred in two patients, whereas 53 patients had reinfection more than 6 months after the previous event. The mean time to reinfection was 3.3 years (± 2.3 years). Repeated IE was more frequently associated with PVE than NVE (*n* = 33, 60% vs. *n* = 22, 40%) but the difference was not significant, (*p* = 0.065). *Staphylococcus aureus* more often caused repeated IE in the NVE group than in the PVE/CIED-IE group (64% vs 15%, *p* < 0.001) whereas enterococci more often caused repeated IE in the PVE/CIED-IE group than in the NVE group (36% vs 9%, *p* = 0.023, supplementary Table 1).

## Discussion

This prospective observational study investigated a large number of IE patients from one out of four Health Regions in Norway. Together with our recent retrospective study from the same region our data provides a complete description of developments in the incidence rates, clinical characteristics and microbiological findings associated with IE in our community over a period of three decades [6]. We found a rising incidence of IE related to prosthetic valves or cardiac implants and in the elderly population, and our data also illustrate a noteworthy increase in enterococcal endocarditis (EE).

Nearly 50% of TAVI-related IE was caused by enterococci, as compared to only 13% caused by *S. aureus*. These numbers differ considerably from those reported in previous studies and recent reviews, where *S. aureus* was reported as the major causative microbe in TAVI-IE, accounting for 20–30% of the cases, closely followed by enterococci, accounting for 13–25% [11, 13–17]. Furthermore, enterococci were the causative bacteria in 26% of our patients with CIED-IE, where the most prevalent microbe was *S. aureus* (40%). Surprisingly, coagulase negative staphylococci accounted for only one case in this group. The relatively high proportion of enterococcal CIED-IE also differed from that reported in previous studies, where *S. aureus* and CoNS were reported to cause as much as 60–70% of the total CIED-infections and enterococci were only identified in approximately 4% [1, 4, 18, 19]. Our EE patients resembled those in previous studies regarding comorbidities, age, and male predominance [3, 15].

An association between urologic or colorectal malignancies and enterococcal bacteriemia as well as an association between urologic procedures and EE has been described [20, 21]. Among our EE patients, a recent urologic procedure was registered for only 7%, and a novel malignancy was only diagnosed in six cases during the diagnostic work-up, three of which were gastrointestinal and only one prostatic. Taken together, we have not been able to identify any plausible underlying reasons for the higher incidence of EE in our material compared to previous studies. Nevertheless, our data strongly support that the empirical antibiotic regimen for suspected IE on TAVI or CIED should include effective coverage of enterococci. Further studies are warranted to identify risk factors for EE on foreign material.

*Staphylococcus aureus* was significantly more often identified in patients with NVE than PVE/CIED-IE (38% vs. 21%, *p* < 0.001). The identification of *S. aureus* in 18% of IE cases on mechanical valves differed from that found in a recent study on PVE in Sweden, where *S. aureus* accounted for 36% of IE on mechanical valves [22]. Although our patient sample was smaller (240 vs. 780 patients) and from a regional study and not nationwide registry, it is interesting to note such differences in microbial epidemiology of PVE between countries with supposedly similar demographics and clinical patient characteristics.

A total of 160 patients received valve replacement surgery. Bacterial DNA was detected in 136 (85%) of the valve biopsies and provided microbial identification for eight culture negative cases. Of the 22 tissue samples with continued per operative growth despite adequate antimicrobial therapy from admission, enterococci were identified in 9 (41%) and S. aureus in 6 (27%), respectively. Clearly, the small numbers do not allow firm conclusions, but we might speculate that this finding reflects enterococci and *S. aureus* as particularly difficult to eradicate from endocardial surfaces and foreign implants due to their capability of biofilm formation, although specific virulence factors for IE in these two bacteria have not yet been identified [23–25].

In our population, repeated endocarditis was observed in 55 patients. Among these, *S. aureus* and enterococci were the most frequent cause of repeated NVE and PVE/CIED-IE, respectively. Relapse of EE has been reported in as much as 10% of the patients, and valve replacement therapy has been found to be a protective factor against one-year relapse and death in this group [26]. Despite a clinical indication for surgery of EE, this is often not performed due to high age, risk, and frailty of the patient [11]. Reinfections with enterococci are frequent but less prevalent in patients treated with combination therapy with amoxicillin-gentamicin or amoxicillin-ceftriaxone compared to monotherapy with ampicillin [26]. Furthermore, a beneficial effect of suppressive antimicrobial treatment for EE has been documented [15]. Based on this, guidelines clarifying the indication for suppressive therapy after the first EE event should be established, as such a treatment option could possibly prevent repeated long-term hospital admissions for old and multimorbid patients.

The highest mortality rates were seen among our cases with IE caused by non-viridans streptococci and *S. aureus* with 30-day mortality rates of 11% and 10%, and 90-day mortality rates of 23% and 16%, respectively. IE caused by non-viridans streptococci and in particular beta-haemolytic streptococci (BHS), have been associated with in-hospital mortality rates between 13–20% in comparable studies [6, 27, 28]. The clinical presentation of IE caused by BHS resemble that of IE caused by *S. aureus,* with more often an initial dysregulated immune response to infection and need for more aggressive therapy and surgery than IE caused by viridans group streptococci [28, 29]. Interestingly, *Streptococcus dysgalacticae* accounted for 51% of IE caused by non-viridans streptococci in our patient cohort. This bacterium is increasingly associated with invasive infections, and is currently the fifth most common cause of bloodstream infections in our region [30].

In our previous study, the mean age of our IE-population increased from 58 to 60 years during the two decades 1996–2005 vs. 2006–2015 whilst 30-day mortality remained 13% and unchanged [6]. In the present study, the mean age increased to 67 years whilst 30-day mortality rate was 9% and lower than expected from comparable populations [31–33]. The reason for this rather striking increase in mean age in only seven years, is not known but noteworthy, as only advanced age remained an independent risk factor for increased 90 days mortality in the fully adjusted model.

We observed annual incidence rates of IE from 10.4 to 14.1 per 100 000 inhabitants per year, with a peak in 2019. Thereafter, a decline was observed in 2020, which we believe was partially associated with the SARS-CoV-2 pandemic. One might assume that the lock-down of the society prevented people from seeking medical care during the first pandemic wave, although Norway had a lower pandemic burden than most other countries. A similar trend for acute hospital admissions has been described in the United Kingdom (UK), although IE-specific data were not presented [34]. Furthermore, remote consulting was widely implemented among general practitioners in the UK in 2020 [35]. This might have led to diagnostic delay in complicated cases, as described in a case report on IE [36]. Our results might suggest that the Covid-19 pandemic influenced the availability of health-care services even in resourceful countries. However, both 30- and 90-day mortality rates were comparable in the period before and after 2019.

A previous study comparing IE in Northern and Southern Europe, documented a significant rise in EE over time, but did not identify major microbiological differences between regions [37]. Whilst IE in high-income countries reflects advances in highly specialized medicine, with an increasing number of patients with intracardial devices, rheumatic heart disease and congenital heart disease are still reported as the most common predisposing condition for IE in developing countries, although documentation is scarce [38, 39].

In the present study, only cases from a limited geographical region in a high-income country with a semi-decentralized structure of the specialist care were included. Therefore, the transferability to other regions might be questioned. Furthermore, this was an observational study without an age- and comorbidity matched control group, thus prohibiting a comparison of overall mortality rates at the end of the follow-up period. Nevertheless, we feel that the study provides important information on the epidemiology, patient characteristics, and microbial aetiology of IE, and will hopefully pave the way for future studies on this important infectious disease.

## Conclusion

The incidence of infective endocarditis increased during the study period, but the mortality rates were relatively low. High age was the only independent risk factor for death within 90 days. *Staphylococcus aureus* was the most frequent bacterial cause overall, but the incidence of EE increased significantly during the study period and was particularly associated with PVE and CIED-IE. Further studies to identify risk factors for EE, and especially in those patients with TAVI and CIED, is warranted.

### Supplementary Information

Supplementary Material 1. Microbial aetiology of repeated IE in western Norway from 2016-2022

## Data Availability

The datasets used and analysed during the current study are available from the corresponding author on reasonable request.

## Abbreviations

IE: Infective endocarditis
CIED: Cardiac implantable electronic devices
EE: Enterococcal endocarditis
Duke-ISCVID: Duke-International Society of Cardiovascular Infectious Diseases
NVE: Native valve endocarditis
PVE: Prosthetic valve endocarditis
TAVI: Transcatheter aortic valve implantation
HUH: Haukeland university hospital
HDH: Haraldsplass deaconess hospital
Maldi-TOF MS: matrix-assisted laser desorption/ionization Time of flight
RNA: Ribonucleic acid
DNA: Deoxyribonucleic acid
PWID: People who inject drugs
S. aureus: Staphylococcus aureus
BHS: Betahaemolytic streptococci
SARS-CoV-2: Severe acute respiratory syndrome coronavirus 2
UK: United Kingdom
