# Supplementary material for: Epidemiological and microbial trends of infective endocarditis in western Norway: a 7-year prospective observational study
Source: BMC Infect Dis. 2024 Jul 17;24:702. doi: 10.1186/s12879-024-09596-3 (PMC11256475; doi:10.1186/s12879-024-09596-3)
Supplement: Supplementary file 1 — Supplementary Material 1. Microbial aetiology of repeated IE in western Norway from 2016-2022 [file 12879_2024_9596_MOESM1_ESM.docx]

**Supplementary table 1 –** Microbial aetiology of repeated IE in western Norway from 2016-2022

| **Variable** | **NVE**  **N=22 (40%)** | **PVE + CIED**  **N=33 (60%)** | **p-value^[[1]](#footnote-1)^** |
| --- | --- | --- | --- |
| *Staphylococcus aureus*, n (%) | 14 (64) | 5 (15) | <0.001 |
| Enterococci, n (%) | 2 (9) | 12 (36) | 0.023 |
| Viridans streptococci, n (%) | 3 (14) | 10 (30) | 0.154 |
| *Strep. dysgalacticae,* n (%) | 2 (9) | 0 (0) | 0.156 |
| Other, n (%) | 0 | 4 (12) | 0.090 |
| No growth, n (%) | 1 (5) | 2 (6) | 0.808 |

*Abbreviations: IE: infective endocarditis, NVE: Native valve endocarditis, PVE: Prosthetic valve endocarditis, CIED: Cardiac implantable electronic devices.*

1. Analysed by Chi-square test or Fisher’s exact test where appropriate. [↑](#footnote-ref-1)
